# Evolving a New Electron Transfer Pathway for Nitrogen Fixation Uncovers an Electron Bifurcating-Like Enzyme Involved in Anaerobic Aromatic Compound Degradation

**DOI:** 10.1128/mbio.02881-22

**Published:** 2023-01-16

**Authors:** Nathan M. Lewis, Abigail Sarne, Kathryn R. Fixen

**Affiliations:** a BioTechnology Institute, University of Minnesota, St. Paul, Minnesota, USA; b Department of Plant and Microbial Biology, University of Minnesota, St. Paul, Minnesota, USA; University of California—Irvine

**Keywords:** nitrogenase, *Rhodopseudomonas palustris*, ferredoxin, NAD^+^-dependent ferredoxin:NADPH oxidoreductase

## Abstract

Nitrogenase is the key enzyme involved in nitrogen fixation and uses low potential electrons delivered by ferredoxin (Fd) or flavodoxin (Fld) to reduce dinitrogen gas (N_2_) to produce ammonia, generating hydrogen gas (H_2_) as an obligate product of this activity. Although the phototrophic alphaproteobacterium Rhodopseudomonas palustris encodes multiple proteins that can reduce Fd, the FixABCX complex is the only one shown to support nitrogen fixation, and R. palustris Fix^–^ mutants grow poorly under nitrogen-fixing conditions. To investigate how native electron transfer chains (ETCs) can be redirected toward nitrogen fixation, we leveraged the strong selective pressure of nitrogen limitation to isolate a suppressor of an R. palustris Δ*fixC* strain that grows under nitrogen-fixing conditions. We found two mutations were required to restore growth under nitrogen-fixing conditions in the absence of functional FixABCX. One mutation was in the gene encoding the primary Fd involved in nitrogen fixation, *fer1*, and the other mutation was in *aadN*, which encodes a homolog of NAD^+^-dependent Fd:NADPH oxidoreductase (Nfn). We present evidence that AadN plays a role in electron transfer to benzoyl coenzyme A reductase, the key enzyme involved in anaerobic aromatic compound degradation. Our data support a model where the ETC for anaerobic aromatic compound degradation was repurposed to support nitrogen fixation in the Δ*fixC* suppressor strain.

## INTRODUCTION

Ferredoxins (Fds) and flavodoxins (Flds) are small protein electron carriers that transfer a single electron from an electron donor to an electron acceptor. In particular, Fds have specialized over evolutionary time to associate with specific partner proteins, allowing Fds to selectively shuttle electrons to specific pathways (1, 2). Key factors such as the structure and charge of the Fd binding surface (3), regulation of Fd abundance (4), and the reduction potential of the Fd (5–7) affect which partner proteins interact with Fd. In theory, these properties can be altered to enable an Fd to interact with a new partner protein(s) to reroute electron flow, but this remains a challenge for rational design since these properties are ill-defined for many Fds. However, Fds can mediate electron transfer in biological reactions essential for growth, making it possible to select mutations that would allow an Fd and a new partner protein to interact (2, 8).

**FIG 1:**
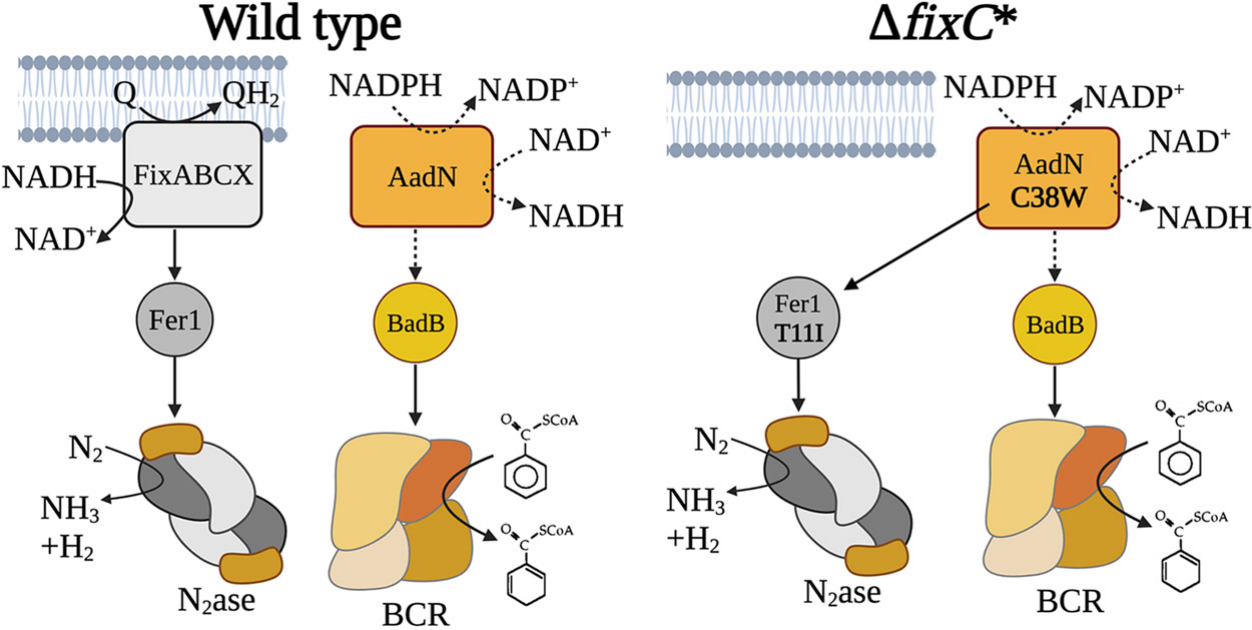
Current model of electron transfer to nitrogenase (N_2_ase) and benzoyl-CoA reductase (BCR) in R. palustris. Electron transfer to BCR is incompatible with nitrogen fixation in the wild type, but the C38W substitution in AadN and T11I substitution in Fer1 enable the electron transfer pathway for benzoate degradation to support nitrogen fixation in ΔfixC*. The hypothesized activity of AadN shown in dotted lines is inferred based on its similarity to NAD^+^-dependent ferredoxin:NADPH oxidoreductase from *Pyrococcus furiosus*.

One such reaction is biological nitrogen fixation, which is catalyzed by the enzyme nitrogenase. Nitrogenase uses large amounts of ATP and low potential electrons delivered by Fd or Fld to reduce atmospheric dinitrogen into ammonia, producing hydrogen gas as an obligate product of this activity (9). In the purple nonsulfur bacterium, Rhodopseudomonas palustris, electron transfer to nitrogenase requires the FixABCX complex, which couples the oxidation of NADH to the reduction of quinone and an Fd or Fld using flavin-based electron bifurcation (FBEB) (Fig. 1) (10–12). R. palustris with a deletion in *fixA*, *fixB*, *fixC*, or *fixX* has a severe growth defect under nitrogen-fixing conditions, despite R. palustris encoding multiple Fd-reducing enzymes that play a role in electron transfer to nitrogenase in other diazotrophs, including pyruvate:Fd oxidoreductase and Fd-NAD(P)^+^ reductase (13–20). R. palustris also encodes six 2[4Fe-4S] Fds, with the primary electron donor to nitrogenase being the Fd, Fer1 (Rpa4631) (11). The Fld, FldA (Rpa2117), can also act as an electron donor in the absence of Fer1 and plays a role under iron-limiting conditions (11). Since R. palustris encodes multiple Fds and Fd-reducing enzymes, R. palustris in which FixABCX is inactive could be leveraged to select for mutations that would enable a new electron transfer chain (ETC) to nitrogenase.

Using an R. palustris Δ*fixC* strain, we isolated a suppressor mutant that restored growth of this strain under nitrogen-fixing conditions. We found that two mutations in the suppressor strain were required to restore nitrogenase activity in the absence of a functional FixABCX complex (Fig. 1). One mutation was in the gene encoding Fer1, while the second was in the uncharacterized gene *rpa0678.* Protein modeling and genetic analysis revealed that the protein encoded by this gene is a homolog of a FBEB NAD^+^-dependent Fd:NADPH oxidoreductase (Nfn), and we found it is required for anaerobic aromatic compound degradation in R. palustris (Fig. 1). Because of its role in anaerobic aromatic compound degradation, we have renamed *rpa0678* to *aadN* for anaerobic aromatic degradation, Nfn-like protein. The data here support a model where a new ETC for nitrogenase formed between components of two endogenous ETCs and provides a system that can be used to study the determinants of selective electron transfer.

## RESULTS

### A mutation in *fer1* improves but is not sufficient for electron transfer to nitrogenase in the absence of FixC.

**FIG 2:**
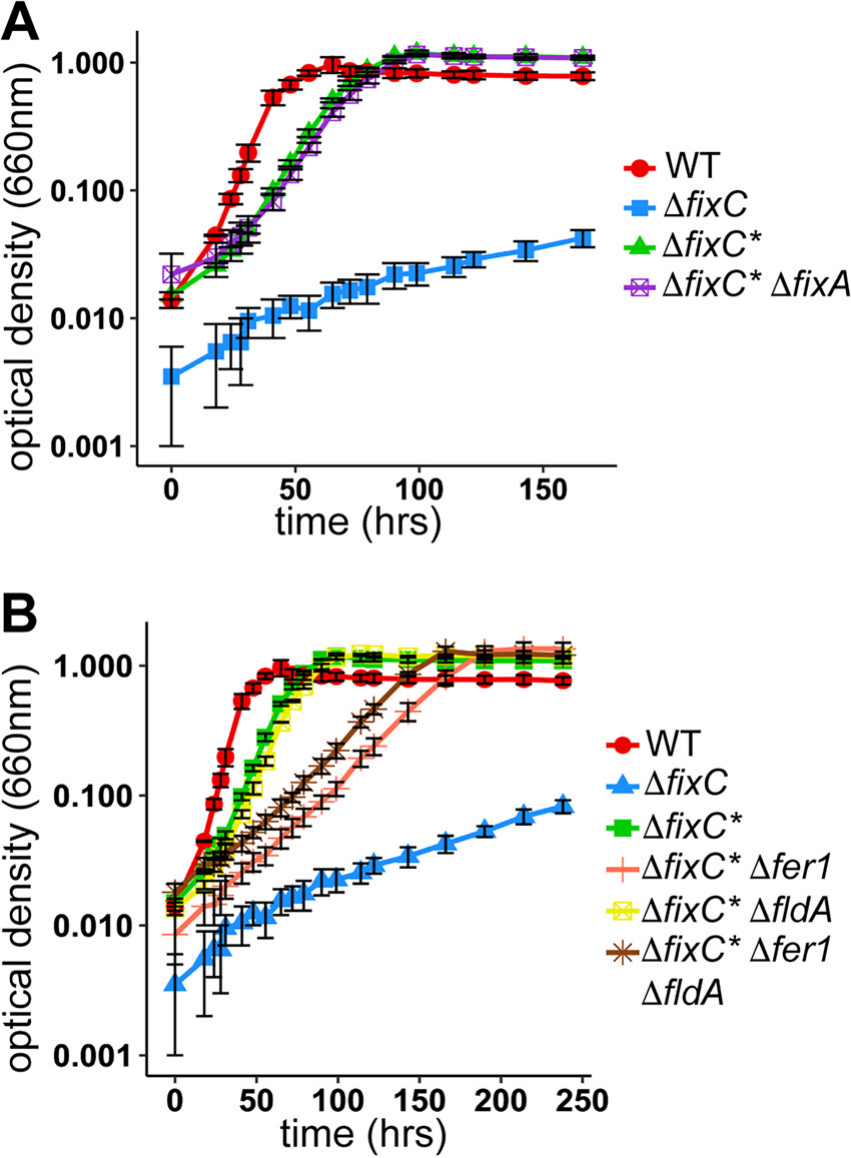
Growth of ΔfixC* in nitrogen-fixing conditions does not require *fixA* but does require *fer1*^T11I^. (A) Growth of wild-type R. palustris CGA753 (WT), R. palustris with a deletion in *fixC* (Δ*fixC*), R. palustris suppressor of Δ*fixC* (ΔfixC*), and R. palustris suppressor of Δ*fixC* with a deletion in *fixA* (ΔfixC* Δ*fixA*) in minimal medium lacking ammonium sulfate (nitrogen-fixing) with 20 mM acetate. (B) Growth of wild-type R. palustris CGA753 (WT), R. palustris with a deletion in *fixC* (Δ*fixC*), R. palustris suppressor of Δ*fixC* (ΔfixC*), R. palustris suppressor of Δ*fixC* with a deletion in *fer1* (ΔfixC* Δ*fer1*), R. palustris suppressor of Δ*fixC* with a deletion in *fldA* (ΔfixC* Δ*fldA*), and R. palustris suppressor of Δ*fixC* with a deletion in *fer1* and *fldA* (ΔfixC* Δ*fer1* Δ*fldA*) in minimal medium lacking ammonium sulfate (nitrogen-fixing) with 20 mM acetate. For both panels A and B, the data are averages of two biological replicates, and error bars represent one standard deviation from the mean.

As shown in Fig. 2A, the R. palustris Δ*fixC* strain (R. palustris Δ*fixC*) has a severe growth defect when grown under nitrogen-fixing conditions, presumably because R. palustris Δ*fixC* is unable to generate enough reduced electron carrier (e.g., Fer1 or FldA) to support nitrogen fixation (10, 11). To select for suppressor mutants of R. palustris Δ*fixC*, this strain was incubated for several weeks under nitrogen-fixing conditions supplemented with 20 mM acetate as a carbon substrate with light provided by a halogen light bulb. One of three replicate liquid cultures grew, from which a suppressor mutant strain of R. palustris Δ*fixC* was isolated, referred to here as R. palustris ΔfixC*. Deletion of *fixA* in R. palustris ΔfixC* did not disrupt the ability of the suppressor strain to grow in nitrogen-fixing conditions, confirming that the remaining Fix complex is not required in R. palustris ΔfixC* (Fig. 2A). Genome sequencing revealed that ΔfixC* accumulated 18 mutations in 16 different genes (Table 1). One of the mutations was in *recQ*, a DNA helicase involved in DNA repair, which may account for the large number of mutations found in the suppressor strain (21). While most of the mutations did not have an obvious connection to electron transfer, one of the mutations identified was in *fer1*, which encodes the primary electron donor to nitrogenase in R. palustris.

**TABLE 1:** Mutations found in the genome of the ΔfixC* strain by whole-genome sequencing

Locus	Gene	Amino acid substitution(s)^*a*^	Gene annotation
RPA0678	*aadN*	C38W	Sulfide dehydrogenase, glutamate synthase
RPA0971	*hupJ*	T76A	Putative hydrogenase expression/formation protein
RPA1135		F224S	Probable ABC transporter
RPA1377		F250Y	Glyoxalase
RPA1470		L138P	Putative dipeptide ABC transporter
RPA1496		Q63stop	Monooxygenase
RPA1542	*bchN*	S63P	Light-independent protochlorophyllide reductase
RPA1975		N277S, M356V	Twin-arginine translocation pathway signal
RPA2153		D230G	Aldehyde dehydrogenase
RPA2193		N152S	Putative ABC transporter
RPA2309		G317fs	Putative iron chelating ABC transporter
RPA3372		A74V	Hypothetical protein
RPA4087		A68fs	ABC-2 type transport system
RPA4534		T15M	Hypothetical protein
RPA4631	*fer1*	T11I	2[4Fe-4S] ferredoxin
RPA4826	*recQ*	A205T	DNA helicase

a “stop” indicates a stop codon. “fs” indicates a frameshift mutation. Only changes that differ from the R. palustris Δ*fixC* parent strain are shown.

The mutation in *fer1* results in the substitution of threonine 11 for isoleucine (T11I). To determine whether the Fer1 T11I variant was required for the suppressor phenotype, the *fer1^T11I^* allele in R. palustris ΔfixC* was replaced with either an in-frame deletion in *fer1* or a wild-type *fer1* allele. Both strains had a significantly lower growth rate than R. palustris ΔfixC* (Table 2; see also Fig. S1 in the supplemental material), indicating that the *fer1^T11I^* allele is required for the suppressor phenotype. However, even in the absence of Fer1, the suppressor strain was still able to grow, albeit at a reduced rate, suggesting that other electron carriers can compensate in the absence of Fer1.

**TABLE 2:** Growth rates of R. palustris strains under nitrogen-fixing conditions

Strain	Avg doubling time (h)^*b*^	Standard deviation
CGA753^*a*^	8	0.2
Δ*fixC*	123	35
ΔfixC*	11	0.1
ΔfixC* Δ*fer1*	37	1.1
ΔfixC* Δ*fldA*	11	0.9
ΔfixC* Δ*fer1* Δ*fldA*	22	0.8
Δ*fixC fer1*^T11I^	61	4.1
Δ*fixC aadN*^C38W^	30	2.4
Δ*fixC fer1*^T11I^ *aadN*^C38W^	18	0.9
ΔfixC* *fer1*^WT^	25	2.5
ΔfixC* *aadN*^WT^	53	3.9

a CGA753 contains a deletion in *anfH* and *vnfH* (see Table S2) and is the parent strain of Δ*fixC*.

b Values are averages of three biological replicates grown in minimal medium lacking ammonium sulfate with 20 mM acetate.

10.1128/mbio.02881-22.1 FIG S1 Repairing mutations in *fer1* or *aadN* in R. palustris ΔfixC* impairs growth under nitrogen-fixing conditions. Growth of R. palustris ΔfixC* (ΔfixC*), R. palustris ΔfixC* with the T11I mutation in *fer1* repaired (ΔfixC* *fer1*^WT^), and R. palustris ΔfixC* with the C38W mutation in *aadN* repaired (ΔfixC* *aadN*^WT^) in minimal medium lacking ammonium sulfate (nitrogen-fixing) with 20 mM acetate provided as a carbon source. The data are averages of three biological replicates, and error bars represent one standard deviation. Download FIG S1, TIFF file, 0.29 MB. Copyright © 2023 Lewis et al. 2023 Lewis et al. https://creativecommons.org/licenses/by/4.0/ This content is distributed under the terms of the Creative Commons Attribution 4.0 International license.

10.1128/mbio.02881-22.6 TABLE S2 All strains, plasmids, and primers used in this study. Download Table S2, DOCX file, 0.02 MB. Copyright © 2023 Lewis et al. 2023 Lewis et al. https://creativecommons.org/licenses/by/4.0/ This content is distributed under the terms of the Creative Commons Attribution 4.0 International license.

**FIG 3:**
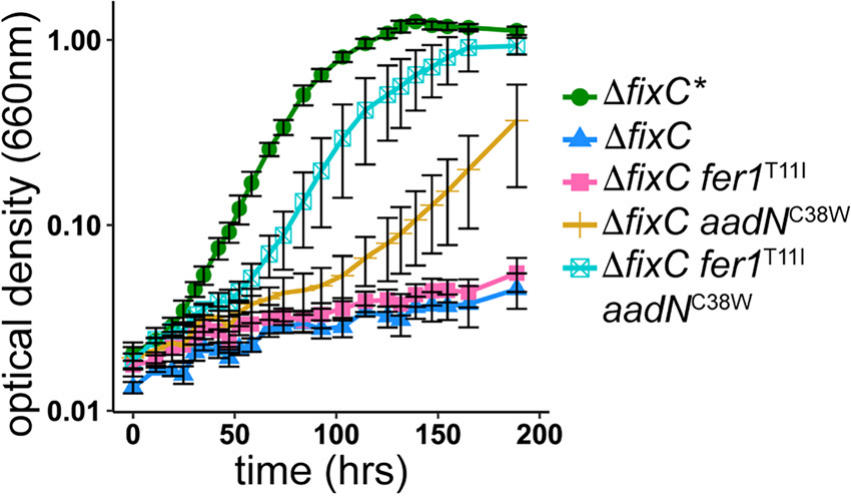
ΔfixC* alleles of *fer1* and *aadN* enable growth of Δ*fixC* in nitrogen fixing conditions. Growth of R. palustris suppressor of Δ*fixC* (ΔfixC*), R. palustris with a deletion in *fixC* (Δ*fixC*), R. palustris Δ*fixC* encoding a *fer1*^T11I^ allele (Δ*fixC fer1*^T11I^), R. palustris Δ*fixC* encoding an *aadN*^C38W^ allele (Δ*fixC aadN*^C38W^), and R. palustris Δ*fixC* encoding the *fer1*^T11I^ and *aadN*^C38W^ alleles (Δ*fixC fer1*^T11I^ *aadN*^C38W^) in minimal medium lacking ammonium sulfate (nitrogen-fixing) supplemented with 20 mM acetate. Data shown are the average of three biological replicates, error bars represent one standard deviation from the mean.

To determine whether the *fer1^T11I^* allele was sufficient to restore growth under nitrogen-fixing conditions in the absence of an intact FixABCX complex, the *fer1^T11I^* allele was introduced into R. palustris Δ*fixC*. As shown in Fig. 3, the *fer1^T11I^* allele did not restore growth in R. palustris Δ*fixC* under nitrogen-fixing conditions, indicating other mutations in R. palustris ΔfixC* are required for electron transfer. This finding revealed that the *fer1*^T11I^ allele was necessary but not sufficient for the suppressor phenotype. Because many of the remaining mutations in R. palustris ΔfixC* had unknown or hypothetical functions (Table 1), we needed to broaden our search for genes involved in electron transfer to nitrogenase in R. palustris ΔfixC*.

Nitrogenase is an energetically expensive reaction, requiring eight low-potential electrons per catalytic cycle (9, 22). We hypothesized that components of the ETC would be transcribed at higher rates to accommodate the demand for reducing power in R. palustris ΔfixC*. Therefore, transcriptome sequencing (RNA-seq) analysis was carried out to compare gene expression changes in R. palustris ΔfixC* compared to wild-type R. palustris under nitrogen-fixing conditions. We found expression of *fldA* had the highest change In gene expression and was upregulated 23-fold in R. palustris ΔfixC* compared to wild-type R. palustris (see Data Set S1 in the supplemental material). To test the role of FldA in the ETC used by R. palustris ΔfixC*, strains were constructed with in-frame deletions in *fldA*, and their growth rates under nitrogen-fixing conditions were measured (Fig. 2B and Table 2). We found FldA was not required for R. palustris ΔfixC* to grow under nitrogen-fixing conditions, and FldA was not redundant with Fer1 in R. palustris ΔfixC* since R. palustris ΔfixC* with a deletion in *fer1* and *fldA* did not grow slower than R. palustris ΔfixC* with a deletion in *fer1* (Fig. 2B). Instead, R. palustris ΔfixC* with a deletion in *fer1* and *fldA* had a slightly higher growth rate compared to R. palustris ΔfixC* with a deletion in *fer1*, suggesting that the presence of FldA may have a slight inhibitory effect in the absence of Fer1 (Table 2). However, this inhibitory effect was not observed when Fer1 is present since the growth rates of R. palustris ΔfixC* and R. palustris ΔfixC* with a deletion in *fldA* were indistinguishable (Table 2). RNA-seq also showed that most other genes encoding enzymes known to reduce Fd were downregulated or showed relatively minor (less than 2-fold) changes in gene expression in R. palustris ΔfixC* (see Table S1).

10.1128/mbio.02881-22.5 TABLE S1 Changes in transcript abundance of genes encoding Fd-reducing enzymes in R. palustris ΔfixC*. Download Table S1, DOCX file, 0.01 MB. Copyright © 2023 Lewis et al. 2023 Lewis et al. https://creativecommons.org/licenses/by/4.0/ This content is distributed under the terms of the Creative Commons Attribution 4.0 International license.

10.1128/mbio.02881-22.8 DATA SET S1 Gene expression changes in R. palustris CGA753 (WT) versus R. palustris ΔfixC* (ΔfixC*) grown in minimal medium without ammonium sulfate (nitrogen-fixing medium) and 20 mM acetate. Download Data Set S1, XLSX file, 0.03 MB. Copyright © 2023 Lewis et al. 2023 Lewis et al. https://creativecommons.org/licenses/by/4.0/ This content is distributed under the terms of the Creative Commons Attribution 4.0 International license.

### A mutation in *aadN* is both necessary and sufficient for electron transfer to nitrogenase in the absence of FixC.

To identify genes required for electron transfer to nitrogenase in R. palustris ΔfixC*, we used a random transposon mutagenesis strategy combined with a metronidazole enrichment (see Fig. S2) (23). Metronidazole is an antibiotic that is activated when reduced by low-potential electron carriers, specifically Fd and Fld, causing cell death (24). Transposon mutants that survive metronidazole enrichment likely have insertions that disrupt electron transfer. Using this approach, we identified one transposon mutant that survived the enrichment and grew similar to R. palustris ΔfixC* under non-nitrogen-fixing conditions but could not grow under nitrogen-fixing conditions. This mutant had a transposon insertion in *aadN* (*rpa0678*). In R. palustris ΔfixC*, *aadN* had a nonsynonymous mutation, encoding a variant of AadN in which cysteine 38 is substituted for tryptophan (C38W) (Table 1). We found that replacing the *aadN*^C38W^ allele in R. palustris ΔfixC* with wild-type *aadN* disrupted the ability of the strain to grow in nitrogen-fixing conditions (Table 2). When the *aadN*^C38W^ mutation was introduced into the parent strain, R. palustris Δ*fixC*, this mutation alone allowed R. palustris Δ*fixC* to grow under nitrogen-fixing conditions, indicating that the *aadN*^C38W^ mutation is both necessary and sufficient to restore growth of R. palustris Δ*fixC* under nitrogen-fixing conditions. However, the growth rate of R. palustris Δ*fixC aadN*^C38W^ was slower than R. palustris ΔfixC* (Fig. 3 and Table 2). When the *aadN*^C38W^ mutation was combined with the *fer1*^T11I^ mutation in R. palustris Δ*fixC*, the growth rate increased (Fig. 3 and Table 2). This suggests that the variants of AadN and Fer1 form a new ETC that can deliver electrons to nitrogenase in the absence of FixABCX (Fig. 1).

10.1128/mbio.02881-22.2 FIG S2 Workflow for transposon mutagenesis with metronidazole enrichment. NFM, nitrogen-fixing medium which is minimal medium lacking ammonium sulfate and 20 mM acetate provided as a carbon source; non-NFM, minimal medium with 20 mM acetate provided as a carbon source; Met, metronidazole; Kan, kanamycin; Tn5 ME, Tn*5* mosaic end. Incubation times are shown in parentheses. Download FIG S2, TIFF file, 2.44 MB. Copyright © 2023 Lewis et al. 2023 Lewis et al. https://creativecommons.org/licenses/by/4.0/ This content is distributed under the terms of the Creative Commons Attribution 4.0 International license.

We measured hydrogen production in growing cultures to quantify nitrogenase activity. Hydrogen is an obligate product of nitrogenase activity (9). Nitrogenase activity can be determined by measuring hydrogen production in these strains because they do not express an uptake hydrogenase, do not encode any other hydrogenases, and accumulate hydrogen only under nitrogen-fixing conditions (25). Introduction of the *aadN*^C38W^ allele into R. palustris Δ*fixC* was sufficient to allow hydrogen production, but it produced about 50% less hydrogen compared to wild-type R. palustris or R. palustris ΔfixC* (Table 3). However, when both the *fer1*^T11I^ and *aadN*^C38W^ alleles were introduced into R. palustris Δ*fixC*, hydrogen production was restored to levels observed for R. palustris ΔfixC*, confirming that only these two mutations are required for electron transfer to nitrogenase in the absence of a functional FixABCX complex (Table 3).

**TABLE 3:** Hydrogen (H_2_) production of R. palustris strains grown under nitrogen-fixing conditions^*a*^

Strain	Allele		Avg H_2_ production (μmol/OD_660_) ± SD^*c*^
	*fer1*	*aadN*	
CGA753^*b*^	Wild type	Wild type	143 ± 12
ΔfixC*	T11I	C38W	114 ± 6.4
Δ*fixC aadN*^C38W^	Wild type	C38W	58 ± 8.0
Δ*fixC fer1*^T11I^ *aadN*^C38W^	T11I	C38W	110 ± 5.6

a All strains were grown in minimal medium lacking ammonium sulfate and supplemented with 20 mM acetate.

b CGA753 contains a deletion in *anfH* and *vnfH* (see Table S2) and is the parent strain of Δ*fixC*.

c Values are averages of three biological replicates calculated by subtracting H_2_ measured from uninoculated samples.

### AadN is a homolog of a Fd-reducing enzyme and is required for anaerobic aromatic compound degradation.

**FIG 4:**
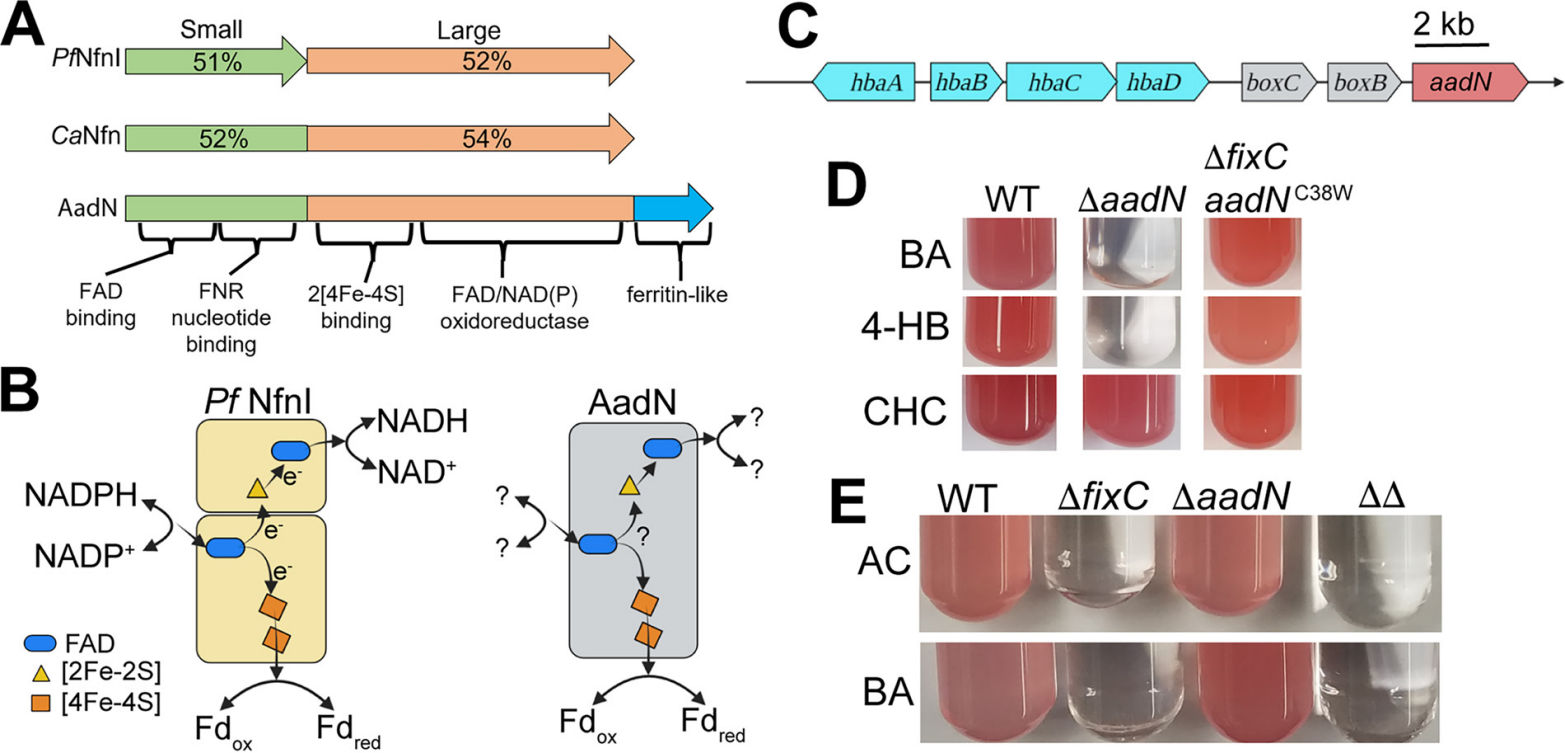
AadN, an Nfn homolog, is required for anaerobic aromatic compound degradation. (A) AadN is homologous to NfnI from *P. furiosus* (*Pf*NfnI) and a pattern B Nfn from *Clostridium autoethanogenum* (*Ca*Nfn). The percent amino acid identity of the large and small subunits of *Pf*NfnI and *Ca*Nfn to AadN are shown over the small (green) and large (orange) regions. (B) *Pf*NfnI ligates two [4Fe-4S] clusters, one [2Fe-2S] cluster, and two flavin adenine dinucleotide (FAD) cofactors per *Pf*NfnI heterodimer. The amino acid sequence of the binding domains for each of these cofactors is conserved in AadN, but it remains unclear if AadN interacts with the same redox pools as *Pf*NfnI. (C) Map of genomic region around *aadN* in R. palustris CGA009 shows that *aadN* is near genes involved in aromatic compound degradation (*hba* genes, cyan). (D) Growth phenotypes of R. palustris CGA753 (WT), R. palustris with a deletion in *aadN* (Δ*aadN*), and R. palustris with a deletion in *fixC* and encoding the *aadN*^C38W^ allele (Δ*fixC aadN*^C38W^) in minimal medium supplemented with 10 mM HCO_3_ and either 5.7 mM benzoate (BA), 5.7 mM 4-hydroxybenzoate (4-HB), or 5.7 mM cyclohexane carboxylate (CHC) as carbon sources. Cultures shown are representative of three independent trials. (E) Growth phenotypes of R. palustris CGA753 (WT), R. palustris with a deletion in *fixC* (Δ*fixC*), R. palustris with a deletion in *aadN* (Δ*aadN*) and R. palustris with a deletion in both *fixC* and *aadN* (ΔΔ) in minimal medium lacking ammonium sulfate (nitrogen-fixing) supplemented with 20 mM acetate (AC) or 5.7 mM benzoate (BA). Cultures shown are representative of three independent trials.

While these data indicate that AadN^C38W^ is required for electron transfer to nitrogenase in R. palustris ΔfixC*, the native function of AadN in R. palustris was unclear. To gain insight into the role of AadN in electron transfer, we used protein modeling to make predictions about the structure and activity of AadN (26, 27). Although AadN is annotated as a sulfide dehydrogenase, sequence analysis of AadN revealed that it shares homology with both the large and small subunit of the enzyme NfnI from *Pyrococcus furiosus* (*Pf*NfnI, Fig. 4A) (28). *Pf*NfnI is an NAD^+^-dependent Fd:NADPH oxidoreductase (Nfn) that uses FBEB to balance NADP(H), NAD(H) and Fd pools to conserve energy and maintain redox balance (Fig. 4B) (28, 29). While the large and the small subunit of *Pf*NfnI are encoded by two separate genes, these subunits are fused in AadN (Fig. 4A) (30). We found the cofactor binding domains in *Pf*NfnI are conserved in AadN and both the large and small subunits share 51% or more amino acid identity (Fig. 4A). This suggests that AadN may be able to carry out FBEB and use electrons from NAD(P)H to reduce Fd (Fig. 4B).

We also found that *aadN* is adjacent to genes involved in anaerobic degradation of aromatic compounds such as benzoate and 4-hydroxybenzoate (4-HB) (Fig. 4C) (31). Anaerobic degradation of these compounds requires the enzyme benzoyl coenzyme A (benzoyl-CoA) reductase, which carries out ATP-dependent electron transfer from a low potential Fd to reduce the aromatic ring of benzoyl-CoA to cyclohex-1,5-diene-1-carbonyl-CoA (see Fig. S3) (32–34). In Thauera aromatica, benzoyl-CoA reductase is supplied with reducing power through a Fd:2-oxoglutarate oxidoreductase known as KorAB (35). While two strains of R. palustris encode *korAB* homologs in the benzoate degradation gene cluster, seven R. palustris strains that encode genes for anaerobic aromatic compound degradation lack *korAB* (36). If AadN plays a role in electron transfer to benzoyl-CoA reductase, we reasoned that strains lacking *korAB* should encode *aadN.* Among R. palustris strains, *aadN* is present in the genomes of the six strains lacking *korAB* but is not found in the two strains encoding *korAB* (Table 4). This suggests that R. palustris strains either use AadN or KorAB for electron transfer during aromatic compound degradation but not both (Table 4). The only R. palustris strain lacking *aadN* and *korAB* was strain HaA2, which cannot degrade aromatic compounds (36).

**TABLE 4:** Genetic distribution of *aadN* and *korAB* among R. palustris strains

R. palustris strain	*korAB* ^*a*^	*aadN* ^*b*^
CGA009	−	+
TIE-1	−	+
DX-1	−	+
DCP3	−	+
BisB18	−	+
PS3	−	+
YSC3	−	+
BisB5	+	−
BisA53	+	−
HaA2^*c*^	−	−

a +, contains a homolog of *korA* (WP_011501953.1) from R. palustris BisB5 with >80% amino acid identity within the anaerobic aromatic degradation gene cluster; −, no homolog present.

b +, contains a homolog of *aadN* (WP_011156245) with >90% amino acid identity within the anaerobic aromatic degradation gene cluster; −, no homolog present.

c R. palustris strain HaA2 is unable to degrade aromatic compounds.

10.1128/mbio.02881-22.3 FIG S3 Degradation pathway of benzoate, 4-hydroxybenzoate (4-HBA), and cyclohexane carboxylate (CHC) in R. palustris incorporating the proposed activity of AadN. The activity of AadN is inferred based on its similarity to *Pf*NfnI. The reaction marked by the dotted line represents the uncertainty of whether benzoyl-CoA reductase (BadDEFG) catalyzes a second reduction of the ring structure after the initial de-aromatization step. Download FIG S3, TIFF file, 0.3 MB. Copyright © 2023 Lewis et al. 2023 Lewis et al. https://creativecommons.org/licenses/by/4.0/ This content is distributed under the terms of the Creative Commons Attribution 4.0 International license.

Given its proximity to genes required for anaerobic aromatic compound degradation, its similarity to *Pf*NfnI, and its importance in electron transfer to nitrogenase in ΔfixC*, we hypothesized that AadN plays a role in electron transfer to benzoyl-CoA reductase. To test this, we looked at the ability of R. palustris Δ*aadN* to metabolize aromatic compounds. Benzoate and 4-HB are converted to benzoyl-CoA and are reductively de-aromatized by benzoyl-CoA reductase, but cyclohexane carboxylate (CHC) enters the same degradation pathway after this de-aromatization step (see Fig. S3) (36). When benzoate or 4-HB were provided as sole carbon sources, R. palustris Δ*aadN* had a growth defect, indicating that *aadN* is required for degradation of benzoate and 4-HB (Fig. 4D). We found that AadN is not required to grow on CHC because both wild-type R. palustris and R. palustris Δ*aadN* were able to grow when CHC was provided as the sole carbon source (Fig. 4D). We also found that the C38W substitution in AadN did not disrupt the ability of R. palustris *aadN*^C38W^ to grow on aromatic carbon sources (Fig. 4D), and R. palustris Δ*fixC aadN*^C38W^ and R. palustris Δ*fixC fer1*^T11I^ *aadN*^C38W^ grow under nitrogen-fixing conditions with benzoate as a carbon source (see Fig. S4), indicating that the C38W variant is able to support both nitrogen fixation and anaerobic aromatic compound degradation simultaneously. In tandem with evidence from protein sequence analysis, this suggests that AadN plays a role in electron transfer to benzoyl-CoA reductase.

10.1128/mbio.02881-22.4 FIG S4 The *aadN*^C38W^ allele is sufficient to support both nitrogen fixation and anaerobic aromatic compound degradation in the absence of functional FixABCX. Growth phenotypes of R. palustris (WT), R. palustris with a deletion in *fixC* (Δ*fixC*), R. palustris with a deletion in *fixC* encoding the *aadN*^C38W^ allele (Δ*fixC aadN*^C38W^), and R. palustris with a deletion in *fixC* encoding the *fer1*^T11I^ and *aadN*^C38W^ allele (Δ*fixC fer1*^T11I^ *aadN*^C38W^) in minimal medium lacking ammonium sulfate (nitrogen-fixing) supplemented with 20 mM acetate (AC) or 5.7 mM benzoate (BA). The image was obtained once all growing cultures reached stationary phase, and the cultures shown are representative of three independent trials. Download FIG S4, TIFF file, 0.69 MB. Copyright © 2023 Lewis et al. 2023 Lewis et al. https://creativecommons.org/licenses/by/4.0/ This content is distributed under the terms of the Creative Commons Attribution 4.0 International license.

### The native ETC for anaerobic aromatic compound degradation is insulated from nitrogen fixation.

Since R. palustris Δ*fixC* is unable to grow under nitrogen-fixing conditions, the ETC for anaerobic aromatic compound degradation and nitrogen fixation are likely insulated from each other. To probe the insulation of these two ETCs, we grew R. palustris strains with an in-frame deletion in *fixC* or *aadN* under nitrogen-fixing conditions with benzoate as a sole carbon source. We found that although *aadN* was required for growth on benzoate under non-nitrogen-fixing conditions (Fig. 4D), R. palustris Δ*aadN* was able to grow with benzoate as a carbon source under nitrogen-fixing conditions (Fig. 4E), suggesting that FixABCX may be able to complement the loss of *aadN* and support benzoate degradation under nitrogen-fixing conditions. However, R. palustris Δ*fixC* was unable to grow under nitrogen-fixing conditions with benzoate, indicating that the ETC for benzoate degradation cannot sustain electron transfer to nitrogenase (Fig. 4E). These data support a model in which electron transfer for benzoate degradation is insulated from nitrogen fixation, and the C38W substitution in AadN overcomes the apparent insulation to allow AadN to function in electron transfer to nitrogenase.

## DISCUSSION

In this study, we identified mutations that would restore electron transfer to nitrogenase in R. palustris in the absence of FixABCX. We hypothesized that changes in Fd or Fld would be required to alter the flow of electrons in the cell, enabling the formation of a new ETC from existing components. While we found that a single amino acid substitution in Fer1 was important for the new ETC, it was not sufficient to support electron transfer to nitrogenase in the absence of FixABCX. We found a mutation in an Nfn-like gene we termed *aadN* was sufficient for electron transfer, but when combined with the mutation in *fer1*, electron transfer to nitrogenase was more efficient. While changing the properties of a Fd played an important role in making the new ETC more efficient, a single change in a Fd-reducing enzyme had a larger role in the formation of the new ETC. Therefore, it is likely that changes in both the Fd-reducing enzyme and the Fd will be required to optimize electron transfer through engineered pathways.

Our approach also uncovered a new role for an uncharacterized Nfn homolog. Nfn homologs are found in all domains of life, but the physiological role of many of these homologs is unknown (30, 37). Sequence homology revealed that AadN is related to Nfn and is part of an uncharacterized family of Nfn enzymes known as pattern B Nfns, in which the two subunits of Nfn are fused (30). Pattern A Nfns, including *Pf*NfnI, ligate two [4Fe-4S] clusters, one [2Fe-2S] cluster, two FAD cofactors, and have binding sites for NADPH and NAD^+^ (28, 38). We found that each of these substrate and cofactor binding sites were conserved in AadN, suggesting that AadN carries out FBEB using NADPH and NAD^+^ to reduce Fd. However, not all Nfn homologs show Nfn bifurcating activity (39), and further structural and enzymatic analysis will be required to determine whether AadN carries out FBEB using the same substrates as Nfn. We showed that AadN is required for aromatic compound degradation and likely plays a role in electron transfer to benzoyl-CoA reductase. To the best of our knowledge, this is the first proposed role for a pattern B Nfn, and this discovery provides evidence that an Nfn-like enzyme can supply reducing power for anaerobic aromatic compound degradation. Our results also implicate the Nfn enzyme family in electron transfer to nitrogenase. Some diazotrophs do not appear to encode any Fd- or Fld-reducing enzymes known to be involved in electron transfer to nitrogenase (40). Our evidence that an Nfn homolog can supply reducing power to nitrogenase may help illuminate the ETCs for nitrogen fixation in some of these diazotrophic organisms.

The insulation of nitrogen fixation and anaerobic aromatic compound degradation highlights the complicated nature of electron transfer insulation. The key enzyme in anaerobic aromatic compound degradation, benzoyl-CoA reductase, requires low potential electrons delivered by a Fd (41). The benzoate degradation gene cluster in R. palustris encodes a Fd known as BadB, and a homolog of BadB in T. aromatica has been shown to have a very low midpoint potential of −587 mV (31, 34). Based on thermodynamics alone, the ETC for anaerobic aromatic compound degradation is predicted to be compatible with nitrogenase. However, we found that even when grown with benzoate, R. palustris Δ*fixC* could not grow under nitrogen-fixing conditions, indicating that the ETC for benzoyl-CoA reductase cannot support electron transfer to nitrogenase.

The electron transfer insulation we observe could be due, in part, to the inability of BadB to interact with nitrogenase. However, our results indicate that the insulation between these two pathways must also be due to the inability of AadN to reduce Fer1 since an amino acid substitution in AadN restores some activity to nitrogenase and is further facilitated by an amino acid substitution in Fer1. It is unclear how these variants enable electron transfer to nitrogenase, but it is likely that they facilitate interaction between AadN and Fer1. The C38W amino acid substitution in AadN could enable electron transfer to nitrogenase by disrupting posttranslational regulation of AadN, affecting the stability of AadN, or altering the Fd binding site of AadN. The threonine residue at position 11 in Fer1 is adjacent to a cysteine predicted to coordinate one of the [4Fe-4S] clusters in Fer1. Many other low-potential 2[4Fe-4S] Fds encode isoleucine at this position and similar threonine to isoleucine substitutions in other Fds have been shown to lower the reduction potential of Fds (5, 34, 42). This suggests that the T11I substitution in Fer1 lowers its reduction potential, although it is unclear why this would facilitate interaction with AadN. Further characterization of how these amino acid substitutions alter the properties of Fer1 and AadN is needed to understand how these proteins can form a new electron transfer pathway to nitrogenase.

In summary, this study illustrates the potential of using a selection strategy to enable a new electron transfer pathway to nitrogenase. Given that electron transfer to nitrogenase is a major hurdle to engineering non-nitrogen-fixing organisms to fix nitrogen, this approach could be useful in evolving nonnative ETCs to be compatible with nitrogenase.

## MATERIALS AND METHODS

### Reagents, bacteria, and culture methods.

All R. palustris strains were grown in defined mineral medium (non-nitrogen-fixing medium) containing 12.5 mM Na_2_HPO_4_, 12.5 mM KH_2_PO_4_, 7.6 mM (NH_4_)_2_SO_4_, 0.1 mM Na_2_S_2_O_3_·5H_2_O, 0.015 mM *p*-aminobenzoic acid, and 1% of a mineral salt solution (see Table S3) (43). Mineral medium lacking ammonium sulfate was used for nitrogen-fixing conditions. Media were prepared using an anaerobic chamber (atmosphere: 98% N_2_, 2% H_2_, <10 ppm O_2_) as described previously (11). Liquid cultures were supplemented with 20 mM acetate, and agar plates were supplemented with 10 mM succinate as carbon sources. Where indicated, liquid cultures were grown with 5.7 mM benzoate, 4-hydroxybenzoate, or cyclohexanecarboxylate and were supplemented with 10 mM HCO_3_ (44). Plates were incubated in GasPak EZ anaerobe container systems at 30°C (Becton Dickinson). Plates were placed within 10 in. of a 60-W light bulb and liquid cultures were placed within 5.5 in. of the light bulb, which provides 30 μmol of photons m^−2^ s^−1^ (General Electric). Where applicable, R. palustris was grown with 100 μg/mL gentamicin and 200 μg/mL kanamycin, and Escherichia coli strains were grown in lysogeny broth at 37°C supplemented with gentamicin (20 μg/mL). For metronidazole enrichment, metronidazole was added to a final concentration of 50 mM.

10.1128/mbio.02881-22.7 TABLE S3 Components of mineral salts solution. Download Table S3, DOCX file, 0.01 MB. Copyright © 2023 Lewis et al. 2023 Lewis et al. https://creativecommons.org/licenses/by/4.0/ This content is distributed under the terms of the Creative Commons Attribution 4.0 International license.

### Genetic manipulation of *R. palustris*.

For each gene of interest, a corresponding pJQ200SK-derived deletion or allelic exchange vector was created (see Table S2) (45). Deletion vectors included ~1 kb of sequence upstream of the start codon and 1 kb of sequence downstream of the stop codon of the gene. Allelic exchange vectors contained 1 kb of sequence upstream and downstream of the point mutation of interest. Construction was carried out as described previously (11). Vectors were mobilized into R. palustris by conjugation using E. coli S17-1 (46). Gene deletions were confirmed by PCR (see Table S2). All allelic exchange strains were confirmed by Sanger sequencing (GENEWIZ, South Plainfield, NJ).

### RNA extraction, cDNA library preparation, and sequencing.

R. palustris cells were harvested from 10 mL of nitrogen-fixing medium after cultures had grown to an optical density (660 nm) of 0.4. Cells were incubated on ice for 10 min and harvested by centrifugation. The cell pellet was frozen in liquid nitrogen and stored at −80°C. Cell pellets were thawed and resuspended in 1 mL of QIAzol lysis reagent (Qiagen, Hilden, Germany) and homogenized using a BioSpec Products BeadBeater-24 (Bartlesville, OK) at maximum rpm for 1 min at 4°C and then allowed to cool for 1 min on ice. This cycle was repeated four times. Total RNA was isolated using the miRNAeasy minikit (Qiagen), and DNA was removed with TURBO DNase (Invitrogen, Carlsbad, CA). RNA was purified and concentrated using the RNeasy MiniElute Cleanup kit (Qiagen). cDNA library construction and library sequencing were performed at GENEWIZ, LLC (South Plainfield, NJ). rRNA was depleted using the Ribo-Zero rRNA removal kit (Illumina, San Diego, CA). cDNA was prepared using the NEBNext Ultra RNA Library Prep kit and sequencing reactions, image analysis, and base calling were performed on an Illumina HiSeq 2500 instrument (Illumina).

### Differential gene expression analysis.

Quality base calling in sequencing data were analyzed using the FastQC application (v 0.11.8; https://www.bioinformatics.babraham.ac.uk/projects/fastqc/) and TrimGalore (v0.6.2) was used to remove adapter sequences, process, and validate all reads using the default parameters. Analysis was performed on the Avadis software package (v3.1.1; Strand Life Sciences, Bengaluru, India). Reads were aligned to the published genome of R. palustris CGA009 and differentially expressed genes were identified using the DESeq2 package (47) in R version 3.6 using the default parameters.

### Transposon mutagenesis and metronidazole enrichment.

Cultures of E. coli BW20767 (48) and R. palustris ΔfixC* were grown to mid-log phase, washed with minimal medium twice, mixed at equivalent concentrations, and then plated on minimal medium agar supplemented with 10 mM succinate, 0.2% yeast extract, and 0.5% Casamino Acids. Plates were incubated overnight at 30°C. After incubation, all visible biomass was transferred from the plate into liquid minimal medium supplemented with 20 mM acetate and kanamycin for 6 h. The Tn*5* mutant pool was then pelleted and transferred to nitrogen-fixing medium with 20 mM acetate and kanamycin overnight. Metronidazole was added, and the culture was allowed to incubate at 30°C for 8 h. The culture was washed with minimal medium twice and plated on minimal medium agar with 10 mM succinate as a carbon source and kanamycin. Roughly 200 individual clones were isolated and screened for their ability to grow under nitrogen-fixing conditions.

### Inverse PCR.

Transposon mutants were grown in liquid minimal medium with 20 mM acetate to stationary phase, and genomic DNA was purified using a Yeast/Bact genomic DNA purification kit (Qiagen). 1 μg of genomic DNA was digested with the restriction enzyme AatII overnight at 37°C to generate fragments of genomic DNA which were, on average, 1,500 bp (New England Biolabs). The digestion product was then treated with Antarctic phosphatase for 1 h at 37°C (New England Biolabs). PCR products were purified using the Zymogen Clean and Concentrator PCR-cleanup kit (Zymo Research, Irvine, CA), and the recovered DNA fragments were ligated together to form closed circular DNA using T4 DNA ligase (New England Biolabs). The library of circular DNA fragments was used as a PCR template with forward and reverse primers specific to the transposable element and amplified using Phusion High-Fidelity DNA polymerase (New England Biolabs). PCR products were separated by electrophoresis on a 1% agarose gel and purified using the Zymoclean Gel DNA recovery kit (Zymo Research, Irvine, CA). The purified DNA fragments were sequenced using Sanger sequencing (GENEWIZ) using primers in (see Table S2).

### Hydrogen measurements.

H_2_ was quantified using a Shimadzu GC-2014 gas chromatogram equipped with a thermal conductivity detector and a 60/80 molecular sieve 5-Å column (6 feet by 1/8 in.; Supelco). H_2_ standards were measured in triplicate. Samples of headspace taken from growing cultures were measured in biological triplicate and technical duplicate. Samples were also taken from the headspace of uninoculated tubes containing the same medium used for growing cultures. The amount of H_2_ found in the headspace of uninoculated tubes was subtracted from the amount of H_2_ measured in growing cultures. For growing cultures, the headspace was sampled at an optical density of 0.4 to 0.55 at 660 nm. Cultures were vortexed briefly before sampling. H_2_ production in each sample was normalized to the optical density (660 nm) of the culture at sampling time.

### Protein sequence analysis.

Specific protein-protein alignments were generated using the Constraint Based Alignment Tool (COBALT; NCBI) using the default parameters. Protein domains were identified using InterPro v.86.0 using the default parameters (26). Homologs of KorAB were identified using JGI/IMG-M using R. palustris BisB5 KorA as a bait sequence. Candidate homologs of *korA* had >80% amino acid identity to KorA and were adjacent to genes involved with anaerobic benzoate or 4-hydroxybenzoate degradation. AadN was used as a bait sequence to identify homologs among the selected R. palustris strains. Homologs of AadN had >90% amino acid identity and were adjacent to anaerobic benzoate or 4-hydroxybenzoate degradation genes.

### Statistical analysis.

Doubling times of different strains in each growth experiment were compared using analysis of variance (ANOVA; *P*_ANOVA_ < 0.001). Welch’s *t* test was used to compare the mean doubling times of individual strains. Similarly, normalized H_2_ accumulation was compared using ANOVA, followed by a Welch’s *t* test. All statistical analyses were performed in R version 4.1.1.

### Data availability.

Genome sequencing data for R. palustris ΔfixC* has been deposited in the NCBI Sequence Read Archive in BioProject PRJNA858464. RNA-seq reads have been deposited on the NCBI Gene Expression Omnibus in BioProject PRJNA858255. KorA and AadN sequences can be found using accession numbers WP_011501953.1 and WP_011156245, respectively.
