# Supplementary figures and images for: Evolving a New Electron Transfer Pathway for Nitrogen Fixation Uncovers an Electron Bifurcating-Like Enzyme Involved in Anaerobic Aromatic Compound Degradation
Source: mBio. 2023 Jan 16;14(1):e02881-22. doi: 10.1128/mbio.02881-22 (PMC9973337; doi:10.1128/mbio.02881-22)

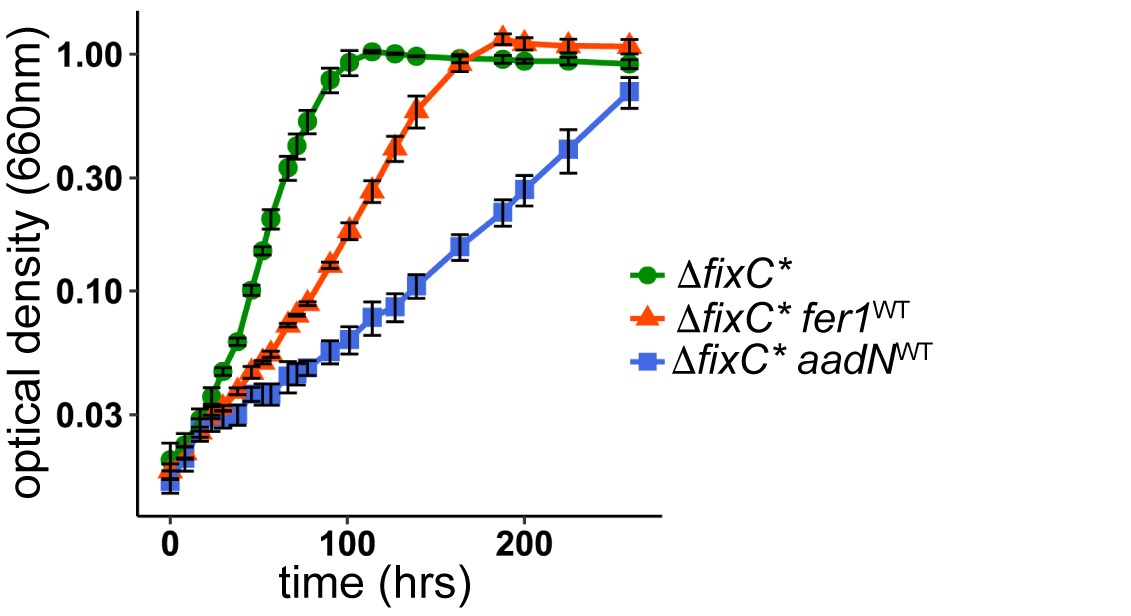

Supplement: FIG S1 [file mbio.02881-22-s0001.tif]

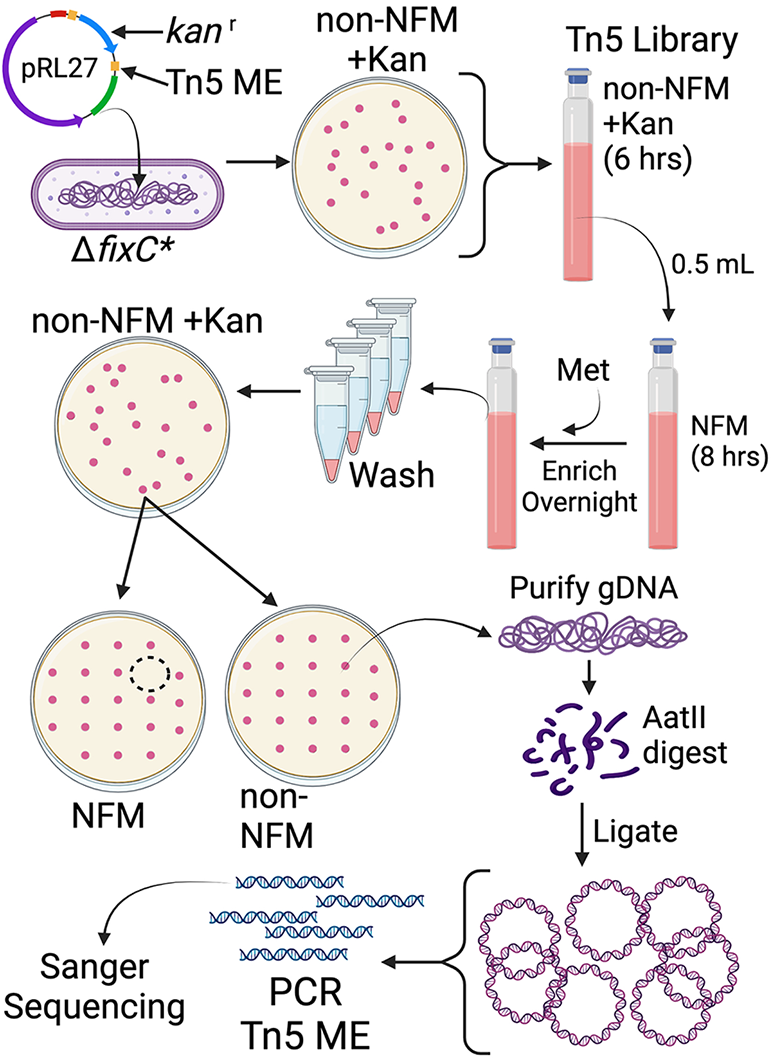

Supplement: FIG S2 [file mbio.02881-22-s0002.tif]

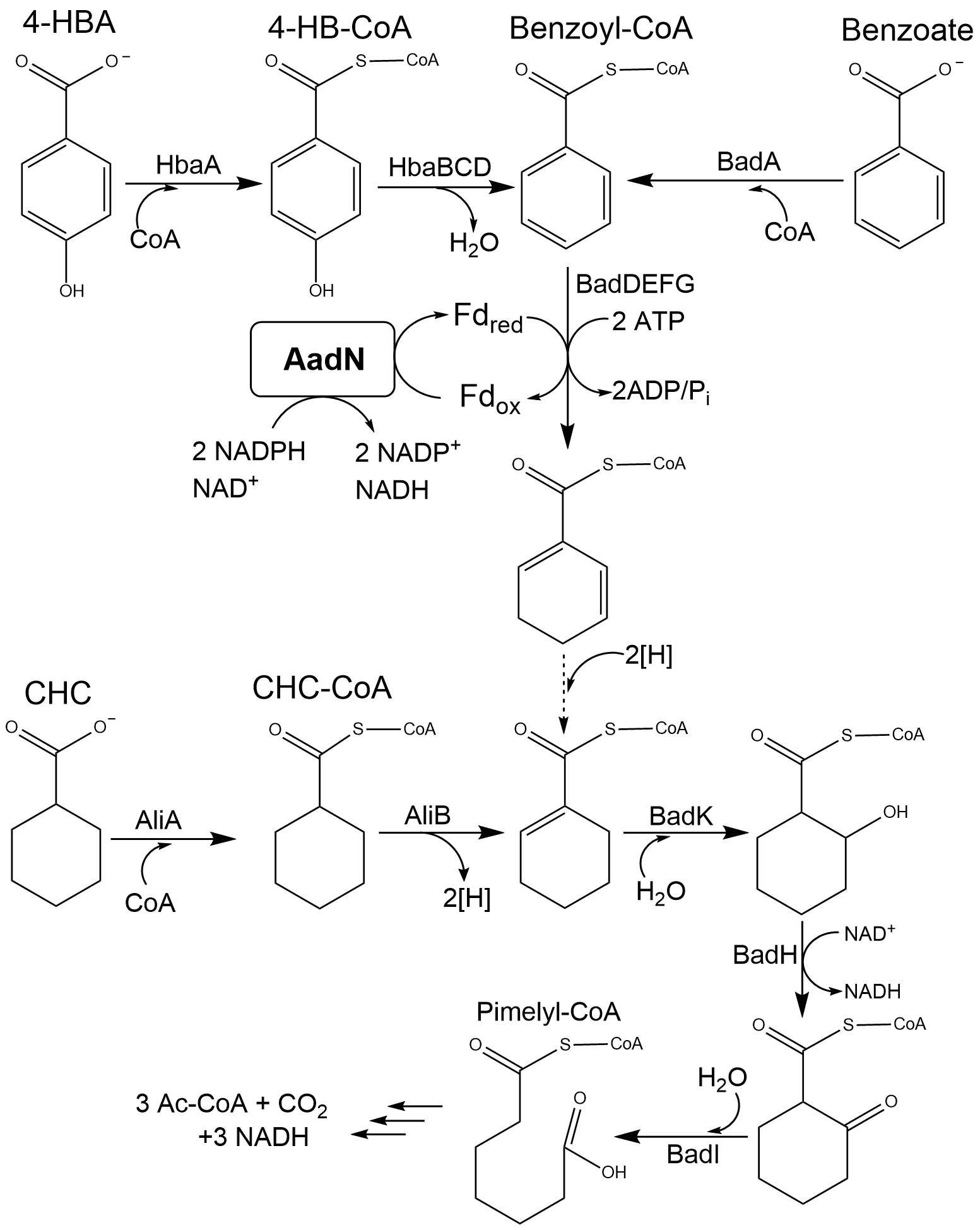

Supplement: FIG S3 [file mbio.02881-22-s0003.tif]

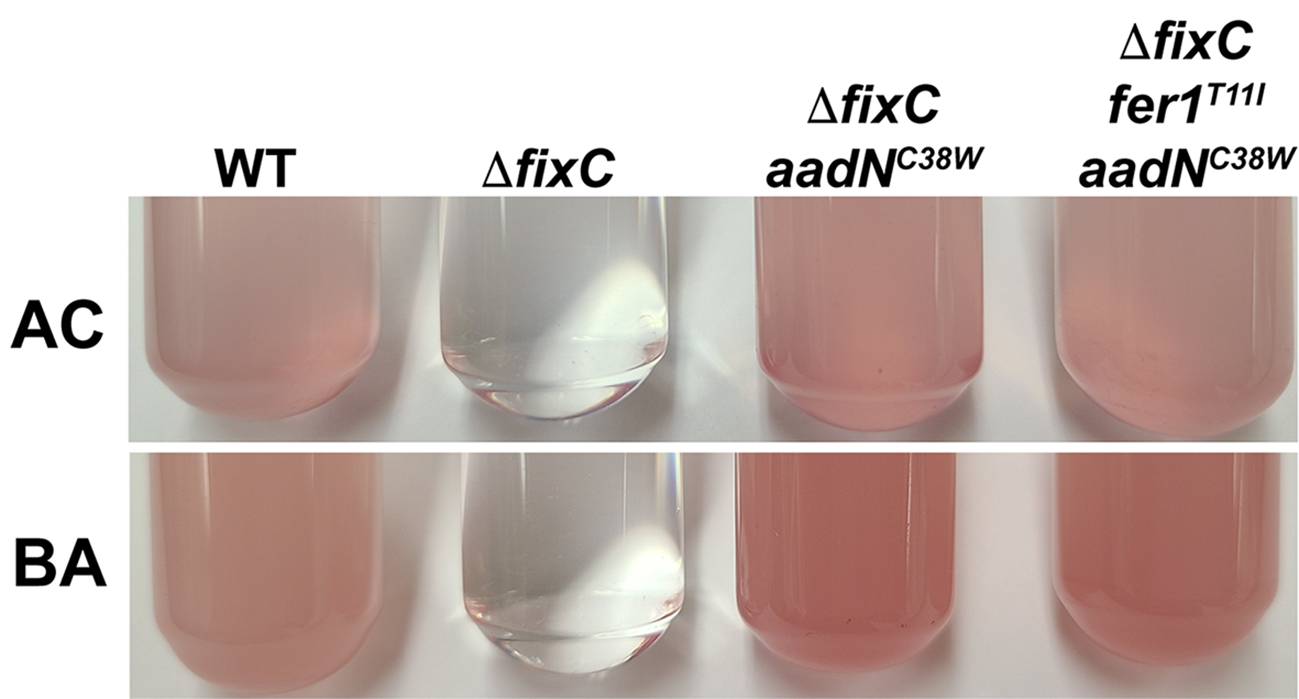

Supplement: FIG S4 [file mbio.02881-22-s0004.tif]
