# Supplementary material for: Evolving a New Electron Transfer Pathway for Nitrogen Fixation Uncovers an Electron Bifurcating-Like Enzyme Involved in Anaerobic Aromatic Compound Degradation
Source: mBio. 2023 Jan 16;14(1):e02881-22. doi: 10.1128/mbio.02881-22 (PMC9973337; doi:10.1128/mbio.02881-22)
Supplement: TABLE S2 [file mbio.02881-22-s0006.docx]

**Table S2.** All strains, plasmids, and primers used in this study.

| **Strain, plasmid, or primer** | **Genotype or primer sequence (5’ – 3’)** | **Reference** |
| --- | --- | --- |
| ***R. palustris* strains** |  |  |
| CGA753 (wild-type) | CGA009 with an in-frame deletion of *vnfH* and *anfH* | (1) |
| Δ*fixC* | CGA753 with an in-frame deletion of *fixC* | (2) |
| Δ*fixC** | Suppressor strain derived from Δ*fixC*. See Table 1 for genotype | This study |
| Δ*fixC** Δ*fixA* | Δ*fixC** with an in-frame deletion of *fixA* | This study |
| Δ*fixC** Δ*fer1* | Δ*fixC** with an in-frame deletion of *fer1* | This study |
| Δ*fixC** Δ*fer1* Δ*fldA* | Δ*fixC** with an in-frame deletion of *fer1, fldA* | This study |
| Δ*fixC** Δ*fer1* Δ*fldA* Δ*ferN* | Δ*fixC** with an in-frame deletion of *fer1, fldA, ferN* | This study |
| Δ*fixC** *aadN*::Tn5 | Δ*fixC** with a Tn5 insertion in codon 38 of *aadN* in the same orientation as the coding sequence | This study |
| Δ*fixC* *fer1*^T11I^ | Δ*fixC* with the Δ*fixC** allele of *fer1* | This study |
| Δ*fixC aadN*^C38W^ | Δ*fixC* with the Δ*fixC** allele of *aadN* | This study |
| Δ*fixC* *fer1*^T11I^ *aadN*^C38W^ | Δ*fixC* with the Δ*fixC** allele of *fer1* and *aadN* | This study |
| Δ*fixC** *fer1*^WT^ | Δ*fixC** with the *fer1*^T11I^ mutation repaired | This study |
| Δ*fixC** *aadN*^WT^ | Δ*fixC** with the *aadN*^C38W^ mutation repaired | This study |
| Δ*aadN* | Mo-only with an in-frame deletion in *aadN* | This study |
| Δ*fixC* Δ*aadN* | Mo-only with an in-frame deletion in *fixC* and *aadN* | This study |
|  |  |  |
| ***E. coli* strains** |  |  |
| S17-1 | *thi pro hdsR hdsM^+^ recA*; chromosomal insertion of RP4-2 (Tc::Mu Km::Tn7) | (3) |
| DH5-α | *fhuA2* Δ*(argF-lacZ)U169 phoA glnV44 Φ80* Δ*(lacZ)M15 gyrA96 recA1 relA1 endA1 thi-1 hsdR17* | NEB |
| BW20767 | RP4–2-Tc::Mu-1 kan::Tn7 integrant leu-63::IS10 *recA1 zbf-5 creB510 hsdR17 endA1 thi uidA* (∆*MluI*)::*pir*+ | (4) |
|  |  |  |
| **Plasmids** |  |  |
| pJQ200SK | Gm^r^, *sacB*; mobilizable suicide vector | (5) |
| pRL27_Tn5 | Conjugation-mobilized suicide vector encoding a Kan^r^ transposable element flanked by Tn5 mosaic ends. | (6) |
| pJQ200SK_*fer1*^T11I^ | pJQ200SK with the sequence amplified by the *rpa4631* F and *rpa4631* R primers from Δ*fixC** genomic DNA inserted at the PstI site | This study |
| pJQ200SK_*fer1*^WT^ | pJQ200SK with the sequence amplified by the *rpa4631* F and *rpa4631* R primers from Mo-only genomic DNA inserted at the PstI site | This study |
| pJQ200SK_*aadN*^C38W^ | pJQ200SK with the sequence amplified by the *aadN* usF and *aadN* 1.3kb R primers from Δ*fixC** genomic DNA inserted at the PstI site | This study |
| pJQ200SK_*aadN*^WT^ | pJQ200SK with the sequence amplified by the *aadN* usF and *aadN* 1.3kb R primers from Mo-only genomic DNA inserted at the PstI site | This study |
|  |  |  |
| **Primers** |  |  |
| *aadN* seqF | GCACAAATCAGCCCATCCGTTGATGG | This study |
| *aadN* seqR | GTTCGTTCGCCTTGTCTTCCTCGGTC | This study |
| *aadN* usF | TCACTAAAGGGAACAAAAGCTGGAGTGCAGCGGACCTGCGACG | This study |
| *aadN* usR | CGCGGGCTTACGTCAGCAGCCCGCGGTGCCGCACACTTGCCG | This study |
| *aadN* dsF | GGGCTCGCCGGCAAGTGTGCGGCACCGCGGGCTGCTGACGTAAGC | This study |
| *aadN* dsR | CCGGGGGATCCACTAGTTCTAGAGCGCACCTTCTGCTCGATCACCGAGC | This study |
| *aadN* 1.3kb_R | CCGGGGGATCCACTAGTTCTAGAGCACGCAGGTCGGTGTGATGCAC | This study |
| pJQ200SK Δ*aadN* F | TGCTCGGTGATCGAGCAGAAGGTGCGCTCTAGAACTAGTGGATCCCCCGG | This study |
| pJQ200SK Δ*aadN* R | GCATCGCGTCGCAGGTCCGCTGCACCCTCCAGCTTTTGTTCCCTTTAGTGAGGG | This study |
| pJQ200SK *aadN* WT::C38W F | TCCAGTGCATCACACCGACCTGCGTGCTCTAGAACTAGTGGATCCCCCGG | This study |
| *rpa4631* seqF | CTTACACCGGCACAGCCCC | This study |
| *rpa4631* F | TCACTAAAGGGAACAAAAGCTGGAGGCGTCTTGCAGGAGCAGGAATTCG | This study |
| *rpa4631* R | CCGGGGGATCCACTAGAGCCCGTGCTCGGCTTCGGAGATC | This study |
| pJQ200SK *rpa4631* F | CGCTGATCTCCGAAGCCGAGCACGGGCTCTAGAACTAGTGGATCCCCCGG | This study |
| pJQ200SK *rpa4631* R | TCGAATTCCTGCTCCTGCAAGACGCCTCCAGCTTTTGTTCCCTTTAGTGAGGG | This study |
| Tn5 seqF | GAGTCAGCAACACCTTCTTCACGAGG | This study |
| Tn5 seqR | GGACAACAAGCCAGGGATGTAACGC | This study |

**SI References**

1. Oda Y, Samanta SK, Rey FE, Wu L, Liu X, Yan T, Zhou J, Harwood CS. 2005. Functional genomic analysis of three nitrogenase isozymes in the photosynthetic bacterium *Rhodopseudomonas palustris*. *J Bacteriol* 187:7784–7794.

2. Huang JJ, Heiniger EK, McKinlay JB, Harwood CS. 2010. Production of hydrogen gas from light and the inorganic electron donor thiosulfate by *Rhodopseudomonas palustris*. *Appl Environ Microbiol* 76:7717–7722.

3. Simon R, Priefer U, Pühler A. 1983. A broad host range mobilization system for *in vivo* genetic engineering: transposon mutagenesis in gram negative bacteria. *Nat Biotechnol* 1:784–791.

4. Metcalf WW, Jiang W, Daniels LL, Kim S-K, Haldimann A, Wanner BL. 1996. Conditionally replicative and conjugative plasmids carrying *lacZα* for cloning, mutagenesis, and allele replacement in Bacteria. *Plasmid* 35:1–13.

5. Quandt J, Hynes MF. 1993. Versatile suicide vectors which allow direct selection for gene replacement in gram-negative bacteria. *Gene* 127:15–21.

6. Larsen R, Wilson M, Guss A, Metcalf W. 2002. Genetic analysis of pigment biosynthesis in *Xanthobacter autotrophicus* Py2 using a new, highly efficient transposon mutagenesis system that is functional in a wide variety of bacteria. *Arch Microbiol* 178:193–201.
