# Supplementary material for: Evolving a New Electron Transfer Pathway for Nitrogen Fixation Uncovers an Electron Bifurcating-Like Enzyme Involved in Anaerobic Aromatic Compound Degradation
Source: mBio. 2023 Jan 16;14(1):e02881-22. doi: 10.1128/mbio.02881-22 (PMC9973337; doi:10.1128/mbio.02881-22)
Supplement: TABLE S1 [file mbio.02881-22-s0005.docx]

**Table S1**. Changes in transcript abundance of genes encoding Fd-reducing enzymes in *R. palustris* Δ*fixC**

| Locus Tag | Gene name | Annotation | Log_2_FC^a^ (Δ*fixC**/WT) | p-value |
| --- | --- | --- | --- | --- |
| RPA0678 | *aadN* | sulfide dehydrogenase, possible glutamate synthase | -0.3 | 0.0067 |
| RPA1228 |  | pyruvate:Fd oxidoreductase | 1.8 | <0.0001 |
| RPA1578 | *cyaA* | Fd:NADP^+^ oxidoreductase | -0.4 | <0.0001 |
| RPA3195 |  | pyruvate:Fd oxidoreductase | 1.5 | <0.0001 |
| RPA3710 |  | Fd:nitrite reductase | -0.8 | <0.0001 |
| RPA4721 |  | pyruvate:Fd oxidoreductase | -0.5 | <0.0001 |
| RPA4722 |  | NAD^+^-dependent Fd:NADPH oxidoreductase | -1.1 | <0.0001 |

^a^ FC: Fold change comparing *R. palustris* Δ*fixC** to wild-type *R. palustris* (WT). “-“ indicates a lower abundance in *R. palustris* Δ*fixC** compared to wild-type.
