# Supplementary material for: Evolving a New Electron Transfer Pathway for Nitrogen Fixation Uncovers an Electron Bifurcating-Like Enzyme Involved in Anaerobic Aromatic Compound Degradation
Source: mBio. 2023 Jan 16;14(1):e02881-22. doi: 10.1128/mbio.02881-22 (PMC9973337; doi:10.1128/mbio.02881-22)
Supplement: TABLE S3 [file mbio.02881-22-s0007.docx]

**Table S3. Components of mineral-salts solution**

| **Mineral-salts solution** | **amount/liter of solution** |
| --- | --- |
| Nitrilotriacetic acid (NTA-free acid) | 20 g |
| MgSO_4_ anhydrous | 28.9 g |
| CaCl_2_⋅2H_2_O | 6.67 g |
| (NH_4_)_6_Mo_7_O_24_⋅4H_2_O | 0.0185 g |
| FeSO_4_⋅7H_2_O | 0.198 g |
| Metal 44 | 100 mL |
| **Metal 44 solution** | **grams/liter of solution** |
| EDTA | 2.5 g |
| ZnSO_4_⋅7H_2_O | 10.95 g |
| FeSO_4_⋅7H_2_O | 5 g |
| MnSO_4_⋅H_2_O | 1.54 g |
| CuSO_4_⋅5H_2_O | 0.392 g |
| Co(NO_3_)_2_⋅6H_2_O | 0.25 g |
| Na_2_B_4_O_7_⋅10H_2_O | 0.177 g |
